# Influence of Personality on mHealth Use in Patients with Diabetes: Prospective Pilot Study

**DOI:** 10.2196/17709

**Published:** 2020-08-10

**Authors:** Jingyuan Su, Michelle Dugas, Xitong Guo, Guodong (Gordon) Gao

**Affiliations:** 1 eHealth Research Institute School of Management Harbin Institute of Technology Harbin China; 2 Center for Health Information & Decision Systems, Department of Decision, Operations, and Information Technologies Robert H Smith School of Business University of Maryland College Park, MD United States

**Keywords:** mHealth, diabetes, adoption, active utilization, personality traits, app

## Abstract

**Background:**

Mobile technology for health (mHealth) interventions are increasingly being used to help improve self-management among patients with diabetes; however, these interventions have not been adopted by a large number of patients and often have high dropout rates. Patient personality characteristics may play a critical role in app adoption and active utilization, but few studies have focused on addressing this question.

**Objective:**

This study aims to address a gap in understanding of the relationship between personality traits and mHealth treatment for patients with diabetes. We tested the role of the five-factor model of personality traits (openness to experience, conscientiousness, extraversion, agreeableness, and neuroticism) in mHealth adoption preference and active utilization.

**Methods:**

We developed an mHealth app (DiaSocial) aimed to encourage diabetes self-management. We recruited 98 patients with diabetes—each patient freely chose whether to receive the standard care or the mHealth app intervention. Patient demographic information and patient personality characteristics were assessed at baseline. App usage data were collected to measure user utilization of the app. Patient health outcomes were assessed with lab measures of glycated hemoglobin (HbA_1c_ level). Logistic regression models and linear regression were employed to explore factors predicting the relationship between mHealth use (adoption and active utilization) and changes in health outcome.

**Results:**

Of 98 study participants, 46 (47%) downloaded and used the app. Relatively younger patients with diabetes were 9% more likely to try and use the app (*P*=.02, odds ratio [OR] 0.91, 95% CI 0.85-0.98) than older patients with diabetes were. Extraversion was negatively associated with adoption of the mHealth app (*P*=.04, OR 0.71, 95% CI 0.51-0.98), and openness to experience was positively associated with adoption of the app (*P*=.03, OR 1.73, 95% CI 1.07-2.80). Gender (*P*=.43, OR 0.66, 95% CI 0.23-1.88), education (senior: *P*=.99, OR 1.00, 95% CI 0.32-3.11; higher: *P*=.21, OR 2.51, 95% CI 0.59-10.66), and baseline HbA_1c_ level (*P*=.36, OR 0.79, 95% CI 0.47-1.31) were not associated with app adoption. Among those who adopted the app, a low education level (senior versus primary *P*=.003; higher versus primary *P*=.03) and a high level of openness to experience (*P*=.048, OR 2.01, 95% CI 1.01-4.00) were associated with active app utilization. Active users showed a significantly greater decrease in HbA_1c_ level than other users (ΔHbA_1c_=−0.64, *P*=.05).

**Conclusions:**

This is one of the first studies to investigate how different personality traits influence the adoption and active utilization of an mHealth app among patients with diabetes. The research findings suggest that personality is a factor that should be considered when trying to identify patients who would benefit the most from apps for diabetes management.

## Introduction

### Diabetes Self-Management

Globally, type 2 diabetes is a common chronic disease, and its incidence is rapidly increasing in countries such as China [1,2]. Treatment for type 2 diabetes is largely self-managed; patients are responsible for engaging in health-promoting behavior on a day-to-day basis [3]. Health-promoting behaviors include dietary control, physical activity, and blood glucose monitoring, and these health-promoting behaviors are often incorporated as essential components of treatment programs in order to keep blood glucose levels within target ranges and to prevent long-term complications [4]. Although long-term self-management and lifestyle behaviors are critical for controlling diabetes, these skills prove difficult for many patients to develop [3].

### Role of mHealth

Mobile technology for health (mHealth) interventions can benefit chronic disease management by delivering real-time monitoring and reminders to a large number of people, enabling the delivery of tailored support and providing low-cost, remote health care services [5]. For diabetes, mHealth interventions are increasingly being used to assist patients with lifestyle changes and health-promoting behaviors [6]. Some recent studies and reviews [7-9] have shown that mHealth smartphone interventions for diabetes self-management have reduced glycated hemoglobin (HbA_1c_) levels and have significantly facilitated self-management for patients; however, the effectiveness of mHealth in improving diabetes outcomes critically depends on voluntary patient engagement. Most studies, in contrast, rely upon randomized trials where patients are exogenously assigned to the mHealth interventions [10]. In many studies that are designed as such, the patients who are required to use the mHealth interventions showed low utilization of the health apps and often had high dropout rates [5,11]. Therefore, within the literature to date, there is a limited understanding of how willing diabetic patients are to adopt mHealth interventions. Within this question, there is also the matter of individual differences that may make patients more or less likely to engage with a given intervention. In order to make use of the full potential of mHealth, researchers, technology companies, and clinicians have been exploring ways of attracting users and increasing mHealth use [12], with the understanding that personality characteristics may be an important consideration when building or recommending different mHealth products [13]. Based on this, we drew on insights from personality research to explore individual differences that could explain heterogeneity in the adoption and active utilization of mHealth to improve diabetes self-management.

### Personality Traits and mHealth Intervention

The five-factor model of personality (also referred to as the big five personality traits) offers a comprehensive framework for examining distinct personal characteristics and their influences. Within the big five, *agreeableness* encompasses the traits of courtesy, cooperation, trust, and tolerance. *Conscientiousness* represents tendencies such as being self-disciplined, organized, and persistent in goal-directed behavior. *Extraversion* is frequently associated with being sociable, gregarious, and optimistic. *Neuroticism* (or emotional instability) is characterized by insecurity, anxiousness, and hostility. *Openness to experience* represents one’s curiosity and willingness to explore new ideas [14]. Taken together, the big five capture the essence of one’s personality.

Past research has recognized that the personal characteristics of users are essential factors in predicting technology adoption and continued utilization of different kinds of apps [15-17]. For example, previous studies have found that more conscientious individuals may be less likely to use leisure apps, but would prefer communication and business apps [18,19]. Openness to experience can be used to predict greater smartphone use and may be positively correlated with the use of social apps [16,20]. Extraverts are more likely to use social networking and instant messaging apps than they are to use apps related to books, references, and education [20]. Individuals with high agreeableness are less likely to use apps related to communication, browser usage, productivity, and gaming [18,19]. Neurotic individuals are likely to adopt apps in general, due to their fastidious and meticulous nature as well as their interest in creative activities [19].

Previous studies [21-25] have also examined the effects of the five personality traits on patient intent to use mHealth apps. Openness to experience had a positive influence on acceptance of an mHealth app for hypertension [21] and was correlated with better adherence to a self-care app for cancer [22]. High conscientiousness was significantly associated with the total number of points earned on an mHealth weight loss app [23]. Gender played a moderating role in the relationship between two specific personality traits, extraversion and emotional stability, and in the behavioral intention to use mHealth apps, in general [24]. Personality traits have been found to influence self-care behavior and glycemic control in patients with type 2 diabetes [25]; however, little is known about how the big five personality traits influence adoption preference and active utilization of diabetic self-management apps. Our exploratory study was designed to help fill this knowledge gap.

### Study Objectives

Using data from a 3-month period, this study explored three relationships: (1) the relationship between the big five personality traits and mHealth adoption, (2) the relationship between the big five personality traits and active utilization of the app, and (3) the effect of mHealth app usage on health outcomes in patients with diabetes. Findings related to the association between personal characteristics and mHealth use in the context of diabetes self-care will contribute to further understanding of the mechanisms underlying high dropout rates and could inform the development of tailored interventions that will engage and improve patient self-management.

## Methods

### Ethical Considerations

The pilot study was approved by the ethics committees at the University of Maryland (1331093-1) and Harbin Institute of Technology School of Management. Informed written consent was obtained from each individual prior to their participation, and patients were provided with complete assurance that all information would be kept confidential. Participation was completely voluntary.

### Recruitment

Patients were recruited through their affiliation with the Endocrinology Department at the Fifth Hospital, Daqing City, China. Daqing has played a key role in diabetic research, as it was the setting for the first batch of studies on diabetes in China, which were well-recognized in journals such as the Lancet [26]. Inclusion criteria for this study were a diagnosis of type 2 diabetes and more than 1 year since that diagnosis. Exclusion criteria were individuals who were blind, deaf, had a diagnosis of serious mental illness (active psychosis, bipolar disorder, schizophrenia, borderline personality disorder, active alcohol addiction, or other), and who lacked a stable residence. 

### Procedure

Eligible patients were identified from electronic medical records and were then notified of the time, address, and content of an upcoming orientation session by phone call.

Patients who were interested in participating attended an orientation session, which included an educational presentation on diabetes by a physician and a detailed description of the study by the research team. In the latter portion of the session, staff explained that patients would choose which treatment they wanted to receive—either the app treatment or standard care—if they chose to participate. The staff then presented the app to explain its functions and why and how it could help the patients. Patients were informed that all participants in both groups would receive the same perks including free HbA_1c_ tests, a portioned dinner plate as a gift, an educational handbook about diabetes self-management, and physician guidance for their health management. They were also informed that, as an additional incentive, those in either group who decreased their HbA_1c_ level during the 3-month period would receive a gift worth 50 RMB (approximately US$ 7). At the end of the orientation session, the prospective participants were entirely free to choose which group to join, and if they chose to participate, they signed a statement to indicate that they were providing informed consent.

Participants attended a second session on the morning following the first session. In the second session, all participants underwent baseline measurement, including HbA_1c_ laboratory tests, and BMI measurements, and completed a questionnaire. Then, participants who chose the mHealth intervention were sent instructions on how to use the app. 

**Figure 1:**
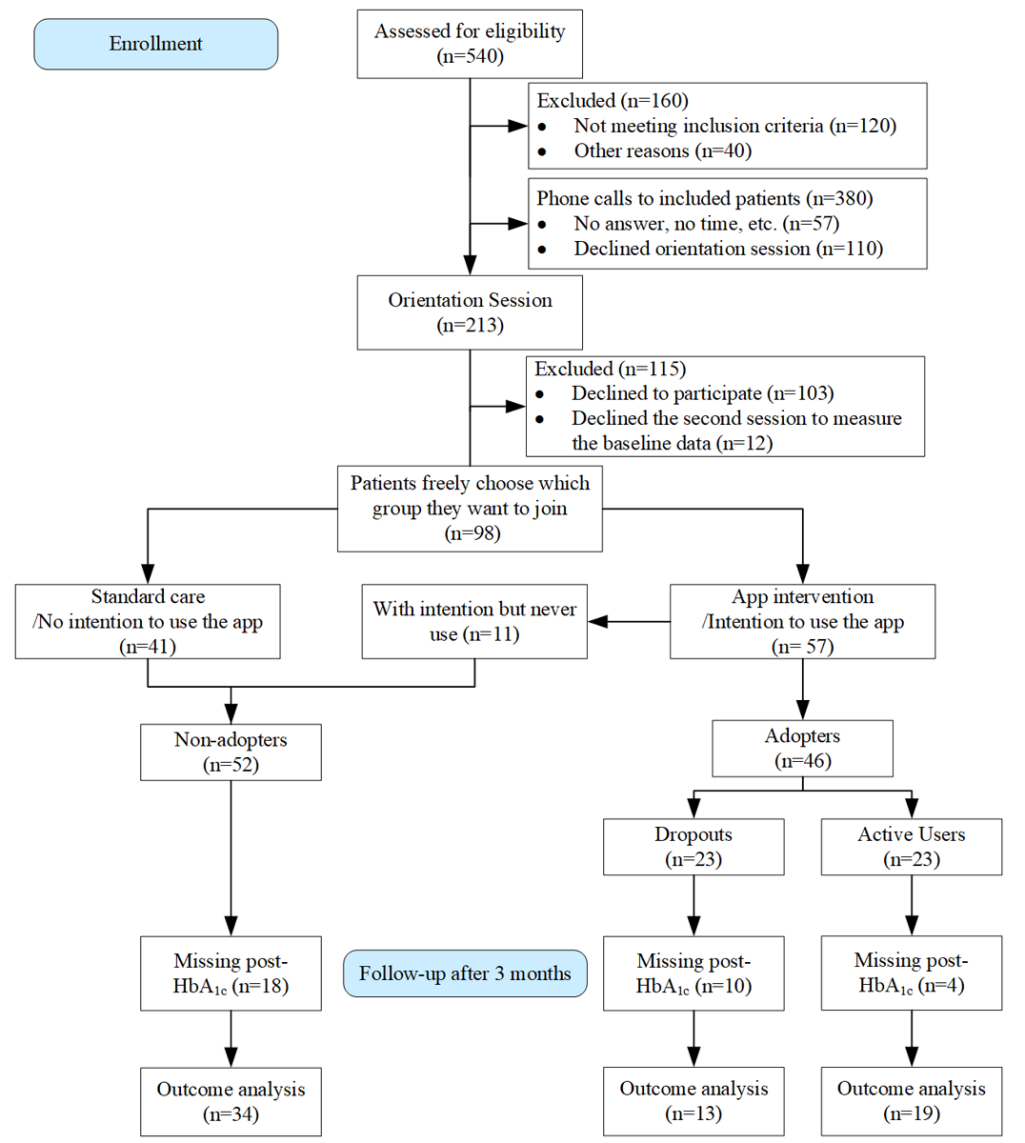
CONSORT flow diagram.

Over the course of 1 month (November 2018), we conducted seven orientation sessions and recruited 98 patients. Participant exclusions and categories are summarized in Figure 1. The study duration was 6 months, but the analyses in this paper reflect midpoint findings (3 months).

### mHealth Intervention

**Figure 2:**
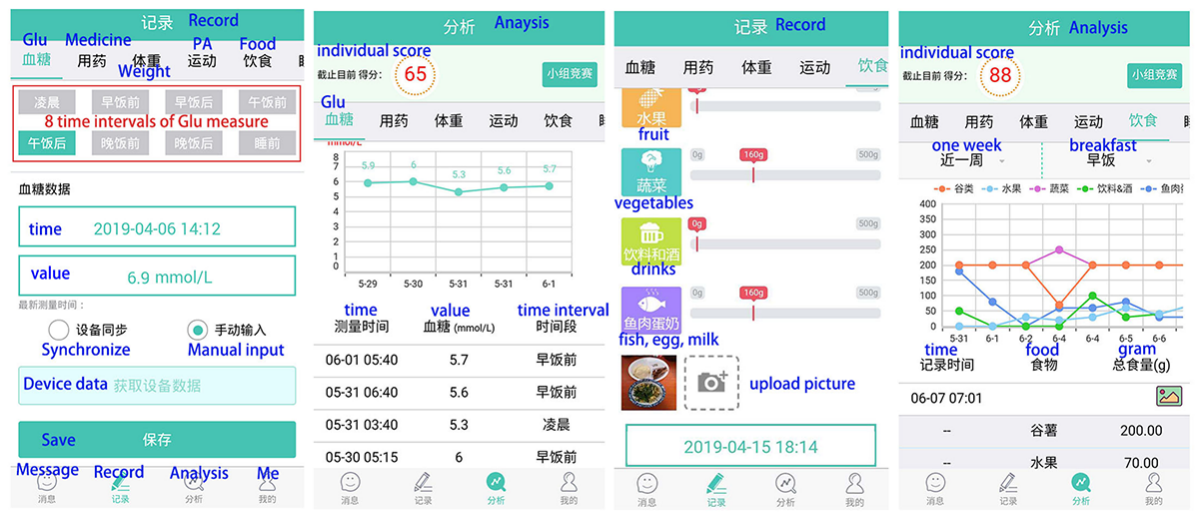
Screen capture images from the DiaSocial app.

Participants who indicated intention to use the app were given access to the health management app (DiaSocial) designed by our research team; screen captured images from the app are shown in Figure 2. The app was available in both iPhone and Android versions. App components consisted of educational resources, tracking features, and feedback. Participants had continuous access to the app throughout the intervention period. Educational information included basic self-management strategies as well as guidance for app use. Participants who used the app were instructed to use it daily and record their progress towards diabetes self-care goals such as exercise, a healthy diet, managing glucose levels, and medication adherence. Physical activity was objectively measured using the participant’s smartphone pedometer or was manually entered by participants into the app. Other records were manually entered.

The app provided feedback through graphical displays of logged behavior. Two types of graphical feedback were provided. The first comprised separate graphs of glucose levels, sleep, medication adherence, diet, weight, and moderate to vigorous intensity physical activity. An information breakdown was provided in the form of daily, weekly, and monthly line charts. The second mode of graphical feedback was a red arrow displayed on the analysis graphs designed to alert participants (1) when blood glucose level prior to meals (breakfast, lunch, or dinner) was greater than 7 mmol/L, (2) when blood glucose level after meals (breakfast, lunch, or dinner) was greater than 10 mmol/L), (3) when BMI was greater than 25 kg/m^2^. 

The app was also gamified such that participants earned scores within the app for use and for reaching target levels of glucose, exercise, nutrition, and medication adherence. The app ranked all the participants according to their daily total scores. At the same time, app users were also assigned to different treatment arms, such as team competition and individual competition, but these experimental conditions did not work out as planned and all analyses were collapsed across the various treatment arms.

### HbA_1c_ Laboratory Test

Glycated hemoglobin (HbA_1c_ level) was chosen as our key clinical outcome because higher HbA_1c_ levels have been associated with more complications and poorer health outcomes [27]. HbA_1c_ level provides a picture of average blood glucose level over a period of months. Patients had blood drawn within 7 days of the study’s launch to assess baseline HbA_1c_ level and 90 days later to determine postintervention HbA_1c_ (post HbA_1c_) levels. Given the 1-month recruitment span, there was variability in the dates on which HbA_1c_ levels were measured. HbA_1c_ values of participants were measured at Daqing Hospital as part of the study, and researchers collected these lab values for analysis.

### Questionnaire Design

The baseline questionnaire was prepared in two languages, English and Chinese, using a semantic translation technique. The content validity of this instrument was assessed by an endocrinologist and also by type 2 diabetic patients to ensure that the questions were appropriate and relevant in the current Chinese setting and culture. The questionnaire items included demographic information (age, gender, education, BMI, and time since diagnosis), measures of health behaviors and attitudes, and a personality inventory.

Personality was assessed with the previously developed Chinese version of the Ten-Item Personality Inventory [28], a widely used, brief measure of the big five personality traits; it has two items per dimension, with each item consisting of a pair of adjectives. Half of the items represent the positive pole of the given dimension and the other half represent the negative pole. Participants rated each item on a 7-point Likert scale, ranging from 1 (disagree strongly) to 7 (agree strongly). A total score for each dimension was generated by transforming the scores of the negative pole items (reverse-scored items), then computing the averaged dimension score. Higher scores indicated a higher level of that personality dimension.

### Statistical Analysis

All analyses were performed with Stata MP (version 15.1; StataCorp LLC) software. Given our research questions, we had three outcome variables of interest: adoption preference (nonadopters=0, adopters=1), app active utilization (dropouts=0, active users=1), and health outcome (ΔHbA_1c_= post HbA_1c_ level – baseline HbA_1c_ level).

We conducted binary logistic regression analyses to determine the association of personality traits with mHealth adoption preference and active utilization. To address our first research question, all patients who agreed to participate in the study were included. To address the second question, the subset of patients who actually used the mHealth app were used in the analysis. An analysis of variance was used for numerical variables and chi-square tests were used for categorical variables to compare the differences between the three types of participants after three months: active users, dropouts, and nonadopters. We also conducted multiple linear regression to explore the relationship between the amount of app usage (number of days per week) and ΔHbA_1c_ for adopters. Analyses included several covariates as well as the five personality traits of interest.

## Results

### Participant Classification and Summary

Based on app usage (average days per week) for each participant (see Multimedia Appendix 1), we defined participant categories as shown in Table 1. Of the patients who agreed to participate (N=98), 58% (57/98) initially indicated their willingness to download and use the app; however, 11 patients never used the app. Nonadopters, therefore, included patients who were not willing to use the app and patients who never used the app even if they initially indicated a willingness to do so. Adopters included dropouts (those who initially used the app, but then became inactive later on) and active users. As defined in Table 1, if an app user did not continue to use the app past the first two months, we regarded that user as a dropout. Users who kept using the app until the third month, were defined as active users. Active users were further divided into 2 groups: low-frequency users (whose weekly average app usage was less than 3.5 days) and high-frequency users (whose weekly average app usage was greater than 3.5 days). Detailed demographics for participant subcategories are shown in Multimedia Appendix 2.

**Table 1:** Definitions of participant categories.

Categories and subcategories				n		Definition	
**Participants**				**98**		**All patients willing to participate in the project.**	
	Intention to use^a^			57		All patients who indicated willingness to use the app at the beginning of the project.	
	**Nonadopters**			**52**			
		No intention to use		41		Patients who were not willing to use the app at the beginning of the project.	
		With intention but never used		11		Patients who indicated willingness to use the app, but then did not use it at all.	
	**Adopters**			**46**			
		Dropouts		23		Patients who used the app, but did not continue use the app in the third month.	
		Active users		23		Patients who continued to use the app into the third month.	
			Low-frequency		14		Patients whose average weekly app usage was less than 3.5 days
			High-frequency		9		Patients whose average weekly app usage was more than 3.5 days

^a^Includes *Nonadopters: With intention but never used*, and *Adopters*.

### App Adoption Preference Among Diabetic Patients and Association With Personality Traits

Table 2 presented the results of the binary logistic regression analyses associating app adoption with sociodemographic and personality traits among diabetic patients. Relatively younger patients with diabetes (mean 56.07, SD 9.06 years) had 9% higher odds of trying the app (*P*=.02, odds ratio [OR] 0.91, 95% CI 0.85-0.98) compared to the odds of older patients with diabetes (mean 62.04, SD 8.04 years). Diabetic patients who were less extraverted had 29% higher odds of trying the app (*P*=.04, OR 0.71, 95% CI 0.51-0.98) compared to the odds of those who were more extraverted. At the same time, diabetic patients who were more open to experience had 73% higher odds of adopting use of the app (*P*=.03, OR 1.73, 95% CI 1.07-2.80) compared to the odds of those who were less openness. Gender (*P*=.43, OR 0.66, 95% CI 0.23-1.88), education (senior: *P*=.99, OR 1.00, 95% CI 0.32-3.11; higher: *P*=.21, OR 2.51, 95% CI 0.59-10.66), and baseline HbA_1c_ level (*P*=.36, OR 0.79, 95% CI 0.47-1.31) were not associated with app adoption. Ordered logistic regression for all participants, which had 5 ordered levels ranging from no intention to use to high-frequency users, also provided similar results and can be found in Multimedia Appendix 3.

**Table 2:** Logistic regression for personality traits associated with adoption preference.

Variables		Participants (N=98)		Logistic regression model				
		Nonadopters (n=52)	Adopters (n=46)	β	OR^a^	95% CI	*P* value	
Age (years), mean (SD)		62.04 (8.04)	56.07 (9.06)	–0.09	0.91	0.85-0.98	.02	
**Gender, n (%)**								
	Female	23 (44)	14 (30)	–0.42	0.66	0.23-1.88	.43	
	Male	29 (56)	32 (70)	N/A^b^	N/A	N/A	N/A	
**Education, n (%)**								
	Primary/junior	23 (44)	10 (22)	N/A	N/A	N/A	N/A	
	Senior/vocational	24 (46)	19 (41)	0.001	1.00	0.32-3.11	.99	
	Higher/university	5 (10)	17 (37)	0.92	2.51	0.59-10.66	.21	
BMI (kg/m^2^), mean (SD)		25.95 (3.15)	25.64 (3.27)	–0.13	0.87	0.74-1.04	.13	
Time since diagnosis (years), mean (SD)		9.52 (8.72)	9.02 (7.02)	0.04	1.04	0.97-1.11	.30	
Baseline HbA_1c_ (%), mean (SD)		7.17 (1.05)	7.14 (0.96)	–0.24	0.79	0.47-1.31	.36	
**Personality traits, mean (SD)**								
	Extraversion	4.97 (1.52)	4.01 (1.81)	–0.34	0.71	0.51-0.98	.04	
	Agreeableness	5.14 (1.12)	5.23 (1.15)	–0.10	0.91	0.55-1.50	.70	
	Conscientiousness	4.42 (1.19)	4.86 (1.32)	0.11	1.11	0.74-1.67	.61	
	Emotional stability	4.29 (1.41)	4.51 (1.52)	0.18	1.20	0.82-1.77	.35	
	Openness	3.99 (1.06)	4.72 (1.30)	0.55	1.73	1.07-2.80	.03	
Constant				8.51			.08	
Chi-square (*df*)				34.1 (12)				<.001

^a^OR: odds ratio.

^b^N/A: not applicable.

### Relationship Between Personality Traits and App Usage Among Diabetic Patients

Table 3 presented the relationship between personality traits and app usage. The results showed that a lower education level—specifically, participants who completed primary school compared to those who had a vocational (senior) or university (higher) education—was associated with greater odds of active app utilization (senior versus primary *P*=.003 and higher versus primary *P*=.03). Higher openness (*P*=.048, OR 2.01, 95% CI 1.01-4.00) was also significantly associated with greater active utilization. We found similar results by using the amount of app usage (number of days per week) as an outcome variable to conduct ordinary least squares linear regression in Multimedia Appendix 4, and by ordered logistic regression in Multimedia Appendix 5.

**Table 3:** Logistic regression for personality traits associated with active utilization.

Variables		Adopters (n=46)		Logistic regression model			
		Dropouts (n=23)	Active users (n=23)	β	OR^a^	95% CI	*P* value
Age (years), mean (SD)		54.96 (8.76)	57.17 (9.80)	0.04	1.04	0.93-1.17	.45
**Gender, n (%)**							
	Female	7 (30)	7 (30)	0.34	1.40	0.17-11.41	.75
	Male	16 (70)	16 (70)	N/A^b^	N/A	N/A	N/A
**Education, n (%)**							
	Primary/junior	1 (4)	9 (39)	N/A	N/A	N/A	N/A
	Senior/vocational	14 (61)	5 (22)	–4.43	0.01	0.001-0.22	.003
	Higher/university	8 (35)	9 (39)	–3.14	0.04	0.003-0.60	.03
BMI (kg/m^2^), mean (SD)		25.96 (3.08)	25.31 (3.49)	–0.12	0.88	0.66-1.19	.41
Time since diagnosis (years), mean (SD)		8.65 (7.07)	9.39 (7.11)	0.04	1.04	0.91-1.18	.57
Baseline HbA_1c_ (%), mean (SD)		7.03 (0.97)	7.25 (0.96)	0.27	1.31	0.50-3.40	.58
**Personality traits, mean (SD)**							
	Extraversion	4.33 (1.90)	3.70 (1.70)	–0.37	0.69	0.38-1.27	.23
	Agreeableness	5.20 (1.04)	5.26 (1.28)	0.56	1.75	0.68-4.52	.25
	Conscientiousness	4.78 (1.41)	4.94 (1.26)	0.06	1.05	0.46-2.40	.92
	Emotional stability	4.74 (1.44)	4.28 (1.59)	–0.17	0.85	0.44-1.63	.62
	Openness	4.46 (1.16)	4.98 (1.41)	0.70	2.01	1.01-4.00	.048
Constant				–2.71			.77
Chi-square (*df*)				23.5 (12)			.02

^a^OR: odds ratio.

^b^N/A: not applicable.

### mHealth App Usage and Patient Health Outcomes

Because some patients did not have their final HbA_1c_ level measured, Table 4 reports descriptive statistics and differences among categories for retained participants after 3 months. Active users showed a greater decrease in HbA_1c_ level (ΔHbA_1c_=–0.64, *P*=.05) than those shown by users in the other groups. We also observed an unexpected decrease in HbA_1c_ level for nonadopters and increase in HbA_1c_ level for dropouts. We observe similar connections between extraversion and openness and health outcomes as we did for overall app adoption—less extraverted participants and those who were more open to experience were more likely to experience a decrease in HbA_1c_ level.

Next, we used linear regression to test the relationship between the amount of app usage (number of days per week) and ΔHbA_1c_ among adopters (Table 5). We found that if app users increased their usage by ten days, their HbA_1c_ level decreased 0.20 points (β=–0.02, *P*=.02) under controlled covariables. More detailed comparisons among all categories are shown in Multimedia Appendix 6.

**Table 4:** Descriptive statistics after 3 months and the differences among categories (N=66).

Variables		Nonadopters (n=34)	Dropouts (n=13)	Active users (n=19)	*F*_2,63_^a^ or chi-square (*df*)^b^	*P* value
Age (years), mean (SD)		63.4 (7.96)	53.23 (9.33)	58.11 (9.98)	6.8^a^	.002
**Gender, n (%)**					**0.7 (2)^b^**	**.70**
	Female	14 (41)	4 (31)	6 (32)		
	Male	20 (59)	9 (69)	13 (68)		
**Education, n (%)**					**17.1 (4)^b^**	**.002**
	Primary/junior	15 (44)	1 (8)	6 (32)		
	Senior/vocational	17 (50)	6 (46)	4 (21)		
	Higher/university	2 (6)	6 (46)	9 (47)		
BMI (kg/m^2^), mean (SD)		26.21 (3.30)	26.72 (3.47)	25.06 (3.68)	1.1^a^	.36
Time since diagnosis (years), mean (SD)		9.32 (8.19)	8.08 (7.71)	8.90 (6.95)	0.1^a^	.89
**Personality traits, mean (SD)**						
	Extraversion	4.47 (1.41)	4.81 (1.38)	3.66 (1.67)	3.8^a^	.03
	Agreeableness	5.03 (1.67)	5.00 (0.89)	5.39 (1.21)	0.8^a^	.48
	Conscientiousness	4.53 (1.22)	4.54 (1.41)	4.89 (1.22)	0.6^a^	.57
	Emotional stability	4.26 (1.49)	4.62 (1.49)	4.36 (1.67)	0.2^a^	.78
	Openness	4.04 (1.13)	4.69 (0.88)	5.03 (1.43)	4.5^a^	.01
Baseline HbA_1c_ (%), mean (SD)		7.22 (1.07)	6.93 (0.89)	7.14 (0.89)	0.5^a^	.64
Post HbA_1c_ (%), mean (SD)		6.97 (1.22)	7.01 (0.81)	6.49 (0.95)	1.7^a^	.18
ΔHbA_1c_ (%), mean (SD)		–0.25 (0.82)	0.08 (0.78)	–0.64 (0.86)	3.1^a^	.050

^a^*F* test was used for numerical variables.

^b^Chi-square was used for categorical variables.

**Table 5:** Model estimates predicting ΔHbA_1c_ for adopters (n=32).

Variables		β	SE	*t* test	*P* value
Age		0.03	0.02	1.69	.11
Female vs male		–0.68	0.47	–1.44	.17
**Education**					
	Senior vs Primary	0.52	0.49	1.07	.30
	Higher vs Primary	0.57	0.46	1.24	.23
BMI		0.03	0.05	0.65	.52
Time since diagnosis		–0.01	0.02	–0.31	.76
Baseline HbA_1c_		–0.32	0.17	–1.87	.08
**Personality traits**					
	Extraversion	–0.02	0.12	–0.17	.87
	Agreeableness	0.09	0.20	0.44	.66
	Conscientiousness	–0.23	0.16	–1.46	.16
	Emotional stability	–0.10	0.12	–0.86	.40
	Openness	0.23	0.14	1.62	.12
Days of app usage		–0.02	0.01	–2.58	.02
Constant		–0.24	3.20	–0.07	.94
Active utilization		0.65			
*F*_13,18_		2.5			.03

## Discussion

### Principal Results

This study suggested a relationship between individual characteristics and mHealth app use (adoption and active utilization) in patients with diabetes. We found that patients with diabetes who were relatively younger, less extraverted, and more open to experience were more likely to adopt use of the mHealth self-management app. In addition, education level and openness to experience were associated with active utilization of the app. Finally, active users were also associated with better clinical outcomes than those of dropouts.

### Comparison With Prior Work

Our finding that relatively younger patients were more likely than older patients to try to adopt the app was in agreement with previous studies [29,30] which found that older adults were less likely to adopt new technology. Older people reported that they do not go online for various reasons including cost, lack of skills, lack of interest, and concerns about information security; however, once older adults did get online, they were just as enthusiastic as younger users [31]. This was also consistent with our own findings, as age was negatively associated with initial adoption, but not with actual use once adopted. In contrast to the outcomes suggested by prior research [32,33], we unexpectedly found that gender, education, and baseline HbA_1c_ were not associated with app adoption.

We also found that mHealth app users with lower education levels may have been less likely to drop out. Low education levels have been associated with inadequate health literacy [34,35], which, in turn, has been associated with lower diabetes-related knowledge and lower engagement in mobile- and web-delivered self-care interventions [36]. This finding may therefore seem surprising; however, Paasche-Orlow et al [37] argued that patients with low health literacy may have difficulty acquiring self-management skills, but once these skills are acquired, they may follow directions more readily than those with higher literacy [37]. In line with this reasoning, our mHealth app provided diabetes self-management educational resources, which may have enabled patients with low education to learn more about diabetes management skills and may have encouraged app usage more than it did for those who already possessed diabetes self-management knowledge [38].

Our results also suggested that extraverted patients with diabetes were less likely to adopt the app, despite the fact that it incorporated and emphasized social features. This may possibly be explained by the fact that the study focused on motivating long-term and continuous self-management, which may not fulfill the social desires of extraverts. Compared to extraverts who prefer to meet friends or participate in social activities to get health support, introverted people may be more likely to use the mHealth app instead of a social network app for this purpose [19]. In fact, a previous study [39] found a similar pattern in which higher extraversion in students was associated with a preference for face-to-face mental health services over eHealth services.

The positive association between openness to experience and patient engagement in mHealth was consistent with the results of previous research [21] on technology adoption. High openness to experience is reflected in curiosity and novelty-seeking—open individuals are willing to try new and different things, are willing to actively seek out new and varied experiences, and value change [15]. At the same time, some research has also found that openness plays an important role in promoting healthy behavior and lowering mortality for patients with chronic disease [40,41]. While mechanisms underlying these findings are not well understood, they are consistent with the notion that openness should be associated with the adoption of novel self-management tools [21] and associated with better health behavior adherence [22]. This means that people more open to experiences may be more likely to adopt and actively use an mHealth app for diabetes management.

The analysis of our findings showed that conscientiousness, agreeableness, and emotional stability did not have a significant relationship with acceptance of the mHealth app. Of particular note, conscientiousness, which reflects self-discipline, did not have a significant role in active app use, which was inconsistent with results of a previous study focusing on a weight loss app [23]. This unexpected finding underscored the need for future research, but differences could be attributed to different population characteristics. Perhaps, our study’s sample of older participants had less prior experience with mHealth apps; therefore, open-mindedness was an especially important predictor of app adoption and active use. There have also been studies [42] suggesting that conscientious patients were more likely to fulfill tasks in accordance with existing strategies, whereas using new technologies could be viewed as more time-consuming and complex than the traditional methods they already use.

Lastly, our pilot study offered support that mHealth improved clinical outcomes of patients with diabetes. This reduction was on the magnitude of a 0.6-point reduction in HbA_1c_ level, which was sizable considering the relatively short intervention period; however, we also noticed that the HbA_1c_ level of participants who dropped out increased while the HbA_1c_ level of nonadopters who never used the app decreased, even though there was no significant difference between the two groups. This may be related to other differences between dropouts and nonadopters; patients with good daily self-management behavior may be less interested in adopting the mHealth app. Meanwhile, patients who had poor self-management behavior may have started using the app to improve their health, yet may not have continuously used the app or followed its guidance, and their HbA_1c_ level may have increased or remained constant. Because this study was not randomized, readers are cautioned against interpreting the results as causal.

### Implications

To the best of our knowledge, this is the first empirical paper to investigate how different personality traits influence the adoption and the active utilization of an mHealth app intervention for patients with diabetes. Our research extends the literature on the adoption of mHealth apps and active utilization to improve diabetes management by exploring the role of personality traits. This work has implications for app designers and practitioners who can leverage this knowledge to target individuals who are most likely to succeed. For instance, patients who are less extraverted and more open to experience may find an app-based intervention the most appealing and effective. With the development of machine learning models, then, an app designer may be able to predict mHealth app user personality traits using easily accessible data and in real time [43,44]. Consequently, designers and practitioners can create or administer personalized mHealth services in a way that enhances patient engagement with helpful apps and which ultimately improves their lifestyle and health.

### Limitations

Caution must be shown in generalizing the findings of this work because it has several limitations, but these limitations also provide opportunities for future research. First, the study used a nonrandomized experimental design in which only those who indicated their willingness to use the mHealth app were placed in the intervention group. While this allowed us to answer research questions about mHealth adoption preference, it made assessment of app efficacy more challenging. In future, a hybrid preference–randomized controlled trial [45] would enable examination of both questions. Second, although all participants, no matter which intervention they chose, were told that those who decrease their HbA_1c_ level during the 3-month period would receive a gift as an incentive, we cannot exclude the possibility that this incentive may have influenced the efficacy of the mHealth app intervention. Building on the encouraging findings from this pilot, future studies may use more rigorous research designs to address this potential impact. Third, as a pilot study, the sample was relatively small in size and came from one hospital in one city. Increasing and diversifying the sample size would provide more confidence that these results are generalizable.

### Conclusions

Although there has been some research about how personality traits impact the use of new technology, there is relatively little that focuses on understanding the impact of individual characteristics, including personality traits, on mHealth app adoption and active utilization among diabetic patients. Our pilot study has made a strong start in addressing this gap by extending the mHealth literature. The study revealed that diabetic patients who are relatively young, introverted, and open to experience were interested in and willing to use the app. Moreover, active use of the app was associated with greater improvements in blood glucose level control. These research findings may have practical effects on the future development of mobile health apps for patients with diabetes.

## Appendix

Multimedia Appendix 1 The weekly days of app usage for each participant with the intention to use.

Multimedia Appendix 2 Detailed descriptive statistics.

Multimedia Appendix 3 Ordered logistic regression for all participants.

Multimedia Appendix 4 Model estimates predicting the days of app usage.

Multimedia Appendix 5 Ordered logistic regression for adopters.

Multimedia Appendix 6 The impact of app usage in the changes in HbA_1c_.

## Abbreviations

HbA_1c_: glycated hemoglobin
ΔHbA_1c_: change in HbA_1c_ level
mHealth: mobile technology for health
OR: odds ratio
RMB: Chinese yuan renminbi

## References

[1] Wang L, Gao P, Zhang M, Huang Z, Zhang D, Deng Q, Li Y, Zhao Z, Qin X, Jin D, Zhou M, Tang X, Hu Y, Wang L (2017). Prevalence and ethnic pattern of diabetes and prediabetes in China in 2013. JAMA.

[2] Jia W, Weng J, Zhu D, Ji L, Lu J, Zhou Z, Zou D, Guo L, Ji Q, Chen L, Chen L, Dou J, Guo X, Kuang H, Li L, Li Q, Li X, Liu J, Ran X, Shi L, Song G, Xiao X, Yang L, Zhao Z, Chinese Diabetes Society (2019). Standards of medical care for type 2 diabetes in China 2019. Diabetes Metab Res Rev.

[3] Ahola AJ, Groop P (2013). Barriers to self-management of diabetes. Diabet Med.

[4] Funnell MM, Anderson RM (2004). Empowerment and self-management of diabetes. Clin Diabetes.

[5] Becker S, Miron-Shatz T, Schumacher N, Krocza J, Diamantidis C, Albrecht U (2014). mHealth 2.0: experiences, possibilities, and perspectives. JMIR Mhealth Uhealth.

[6] Quinn CC, Butler EC, Swasey KK, Shardell MD, Terrin MD, Barr EA, Gruber-Baldini AL (2018). Mobile diabetes intervention study of patient engagement and impact on blood glucose: mixed methods analysis. JMIR Mhealth Uhealth.

[7] Hamine S, Gerth-Guyette E, Faulx D, Green BB, Ginsburg AS (2015). Impact of mHealth chronic disease management on treatment adherence and patient outcomes: a systematic review. J Med Internet Res.

[8] Fu H, McMahon SK, Gross CR, Adam TJ, Wyman JF (2017). Usability and clinical efficacy of diabetes mobile applications for adults with type 2 diabetes: a systematic review. Diabetes Res Clin Pract.

[9] Cui M, Wu X, Mao J, Wang X, Nie M (2016). T2DM self-management via smartphone applications: a systematic review and meta-analysis. PLoS One.

[10] Wu Y, Yao X, Vespasiani G, Nicolucci A, Dong Y, Kwong J, Li L, Sun X, Tian H, Li S (2017). Mobile app-based interventions to support diabetes self-management: a systematic review of randomized controlled trials to identify functions associated with glycemic efficacy. JMIR Mhealth Uhealth.

[11] Krebs P, Duncan DT (2015). Health app use among us mobile phone owners: a national survey. JMIR Mhealth Uhealth.

[12] (2015). Patient Adoption of mHealth. https://www.iqvia.com/-/media/iqvia/pdfs/institute-reports/patient-adoption-of-mhealth.pdf.

[13] Dugas M, Crowley K, Gao GG, Xu T, Agarwal R, Kruglanski AW, Steinle N (2018). Individual differences in regulatory mode moderate the effectiveness of a pilot mHealth trial for diabetes management among older veterans. PLoS ONE.

[14] Digman JM (1990). Personality structure: emergence of the five-factor model. Annu Rev Psychol.

[15] Devaraj S, Easley RF, Crant JM (2008). How does personality matter? relating the five-factor model to technology acceptance and use. Information Systems Research.

[16] Correa T, Hinsley AW, de Zúñiga HG (2010). Who interacts on the web?: the intersection of users’ personality and social media use. Comput Human Behav.

[17] McElroy, Hendrickson, Townsend, DeMarie (2007). Dispositional factors in internet use: personality versus cognitive style. MIS Quarterly.

[18] Stachl C, Hilbert S, Au J, Buschek D, De Luca A, Bischl B, Hussmann H, Bühner M (2017). Personality traits predict smartphone usage. Eur J Pers.

[19] Xu R, Frey RM, Fleisch E, Ilic A (2016). Understanding the impact of personality traits on mobile app adoption – insights from a large-scale field study. Computers in Human Behavior.

[20] Kim Y, Briley DA, Ocepek MG (2015). Differential innovation of smartphone and application use by sociodemographics and personality. Comput Human Behav.

[21] Breil B, Kremer L, Hennemann S, Apolinário-Hagen J (2019). Acceptance of mHealth apps for self-management among people with hypertension. Stud Health Technol Inform.

[22] Mikolasek M, Witt CM, Barth J (2018). Adherence to a mindfulness and relaxation self-care app for cancer patients: mixed-methods feasibility study. JMIR Mhealth Uhealth.

[23] Hales S, Turner-McGrievy GM, Wilcox S, Davis RE, Fahim A, Huhns M, Valafar H (2017). Trading pounds for points: Engagement and weight loss in a mobile health intervention. Digit Health.

[24] Nunes A, Limpo T, Castro S, Bamidis PD, Ziefle M, Maciaszek L (2019). Individual factors that influence the acceptance of mobile health apps: the role of age, gender, and personality traits. Information and Communication Technologies for Ageing Well and e-Health.

[25] Skinner TC, Bruce DG, Davis TME, Davis WA (2013). Personality traits, self-care behaviours and glycaemic control in type 2 diabetes: the Fremantle diabetes study phase ii. Diabet Med.

[26] Li G, Zhang P, Wang J, Gregg EW, Yang W, Gong Q, Li H, Li H, Jiang Y, An Y, Shuai Y, Zhang B, Zhang J, Thompson TJ, Gerzoff RB, Roglic G, Hu Y, Bennett PH (2008). The long-term effect of lifestyle interventions to prevent diabetes in the China Da Qing Diabetes Prevention Study: a 20-year follow-up study. Lancet.

[27] Ye Q, Fu J (2017). Paediatric type 2 diabetes in China-pandemic, progression, and potential solutions. Pediatr Diabetes.

[28] Gosling SD, Rentfrow PJ, Swann WB (2003). A very brief measure of the big-five personality domains. J Res Pers.

[29] Czaja SJ, Charness N, Fisk AD, Hertzog C, Nair SN, Rogers WA, Sharit J (2006). Factors predicting the use of technology: findings from the Center for Research and Education on Aging and Technology Enhancement (CREATE). Psychol Aging.

[30] Andone I, Błaszkiewicz K, Eibes M, Trendafilov B, Montag C, Markowetz A (2016). How age and gender affect smartphone usage. https://dl.acm.org/doi/pdf/10.1145/2968219.2971451.

[31] Fox S (2004). Older Americans and the Internet. https://www.pewresearch.org/internet/wp-content/uploads/sites/9/media/Files/Reports/2004/PIP_Seniors_Online_2004.pdf.pdf#page=9&zoom=auto,-14,733.

[32] Guo X, Han X, Zhang X, Dang Y, Chen C (2015). Investigating m-health acceptance from a protection motivation theory perspective: gender and age differences. Telemed J E Health.

[33] Mackert M, Mabry-Flynn A, Champlin S, Donovan EE, Pounders K (2016). Health literacy and health information technology adoption: the potential for a new digital divide. J Med Internet Res.

[34] van DHI, Wang J, Droomers M, Spreeuwenberg P, Rademakers J, Uiters E (2013). The relationship between health, education, and health literacy: results from the Dutch Adult Literacy and Life Skills Survey. J Health Commun.

[35] Schillinger D, Barton LR, Karter AJ, Wang F, Adler N (2016). Does literacy mediate the relationship between education and health outcomes? a study of a low-income population with diabetes. Public Health Rep.

[36] Chen Q, Carbone ET (2017). Functionality,implementation, impact, and the role of health literacy in mobile phone apps for gestational diabetes: scoping review. JMIR Diabetes.

[37] Paasche-Orlow MK, Schillinger D, Greene SM, Wagner EH (2006). How health care systems can begin to address the challenge of limited literacy. J Gen Intern Med.

[38] von Wagner C, Steptoe A, Wolf MS, Wardle J (2008). Health literacy and health actions: a review and a framework from health psychology. Health Educ Behav.

[39] March S, Day J, Ritchie G, Rowe A, Gough J, Hall T, Yuen CYJ, Donovan CL, Ireland M (2018). Attitudes toward e-mental health services in a community sample of adults: online survey. J Med Internet Res.

[40] Jackson JJ, Hill PL, Payne BR, Roberts BW, Stine-Morrow EAL (2012). Can an old dog learn (and want to experience) new tricks? cognitive training increases openness to experience in older adults. Psychology and Aging.

[41] Jonassaint CR, Boyle SH, Williams RB, Mark DB, Siegler IC, Barefoot JC (2007). Facets of openness predict mortality in patients with cardiac disease. Psychosom Med.

[42] Shambare N (2013). Examining the Influence of Personality Traits on Intranet Portal Adoption by Faculty in Higher Education. Ph.D. Dissertation, Northcentral University.

[43] Azucar D, Marengo D, Settanni M (2018). Predicting the big 5 personality traits from digital footprints on social media: a meta-analysis. Pers Individ Dif.

[44] Mønsted B, Mollgaard A, Mathiesen J (2018). Phone-based metric as a predictor for basic personality traits. Journal of Research in Personality.

[45] Almeida FA, Pardo KA, Seidel RW, Davy BM, You W, Wall SS, Smith E, Greenawald MH, Estabrooks PA (2014). Design and methods of “diaBEAT-it!”: A hybrid preference/randomized control trial design using the RE-AIM framework. Contemporary Clinical Trials.

