# Supplementary material for: Influence of Personality on mHealth Use in Patients with Diabetes: Prospective Pilot Study
Source: JMIR Mhealth Uhealth. 2020 Aug 10;8(8):e17709. doi: 10.2196/17709 (PMC7445619; doi:10.2196/17709)
Supplement: Multimedia Appendix 1 [file mhealth_v8i8e17709_app1.docx]

**Multimedia Appendix 1**

Table. The weekly days of app usage for each participant with intention to use (n=57).

| ID | Level A ^a^ | Week | | | | | | | | | | | | Total days | Mean days ^b^ | Total Behavior ^c^ |
| --- | --- | --- | --- | --- | --- | --- | --- | --- | --- | --- | --- | --- | --- | --- | --- | --- |
|  |  | 1 | 2 | 3 | 4 | 5 | 6 | 7 | 8 | 9 | 10 | 11 | 12 |  |  |  |
| 9159 | 1 | 1 | 0 | 0 | 0 | 0 | 0 | 0 | 0 | 0 | 0 | 0 | 0 | 1 | 0.08 | 1 |
| 9073 | 1 | 1 | 0 | 0 | 0 | 0 | 0 | 0 | 0 | 0 | 0 | 0 | 0 | 1 | 0.08 | 1 |
| 9059 | 1 | 1 | 0 | 0 | 0 | 0 | 0 | 0 | 0 | 0 | 0 | 0 | 0 | 1 | 0.08 | 1 |
| 8916 | 1 | 1 | 0 | 0 | 0 | 0 | 0 | 0 | 0 | 0 | 0 | 0 | 0 | 1 | 0.08 | 1 |
| 8165 | 1 | 1 | 0 | 0 | 0 | 0 | 0 | 0 | 0 | 0 | 0 | 0 | 0 | 1 | 0.08 | 1 |
| 7555 | 1 | 1 | 0 | 0 | 0 | 0 | 0 | 0 | 0 | 0 | 0 | 0 | 0 | 1 | 0.08 | 1 |
| 5967 | 1 | 1 | 0 | 0 | 0 | 0 | 0 | 0 | 0 | 0 | 0 | 0 | 0 | 1 | 0.08 | 1 |
| 5375 | 1 | 1 | 0 | 0 | 0 | 0 | 0 | 0 | 0 | 0 | 0 | 0 | 0 | 1 | 0.08 | 1 |
| 5102 | 1 | 1 | 0 | 0 | 0 | 0 | 0 | 0 | 0 | 0 | 0 | 0 | 0 | 1 | 0.08 | 1 |
| 4074 | 1 | 1 | 0 | 0 | 0 | 0 | 0 | 0 | 0 | 0 | 0 | 0 | 0 | 1 | 0.08 | 1 |
| 2075 | 1 | 1 | 0 | 0 | 0 | 0 | 0 | 0 | 0 | 0 | 0 | 0 | 0 | 1 | 0.08 | 1 |
| 7794 | 2 | 1 | 0 | 0 | 0 | 0 | 1 | 2 | 1 | 0 | 0 | 0 | 0 | 5 | 0.42 | 10 |
| 7475 | 2 | 4 | 1 | 0 | 0 | 0 | 0 | 0 | 0 | 0 | 0 | 0 | 0 | 5 | 0.42 | 9 |
| 2653 | 2 | 3 | 2 | 0 | 0 | 0 | 0 | 0 | 0 | 0 | 0 | 0 | 0 | 5 | 0.42 | 12 |
| 4958 | 2 | 2 | 0 | 3 | 0 | 0 | 0 | 0 | 0 | 0 | 0 | 0 | 0 | 5 | 0.42 | 9 |
| 3486 | 2 | 1 | 1 | 2 | 0 | 1 | 0 | 0 | 0 | 0 | 0 | 0 | 0 | 5 | 0.42 | 9 |
| 9193 | 2 | 2 | 1 | 2 | 0 | 0 | 0 | 0 | 0 | 0 | 0 | 0 | 0 | 5 | 0.42 | 10 |
| 1670 | 2 | 2 | 0 | 5 | 0 | 3 | 0 | 0 | 0 | 0 | 0 | 0 | 0 | 10 | 0.83 | 23 |
| 4135 | 2 | 1 | 2 | 0 | 1 | 0 | 0 | 0 | 0 | 0 | 0 | 0 | 0 | 4 | 0.33 | 6 |
| 4901 | 2 | 4 | 0 | 1 | 1 | 0 | 0 | 0 | 0 | 0 | 0 | 0 | 0 | 6 | 0.50 | 12 |
| 4102 | 2 | 1 | 1 | 2 | 1 | 0 | 0 | 0 | 0 | 0 | 0 | 0 | 0 | 5 | 0.42 | 25 |
| 1316 | 2 | 2 | 3 | 4 | 1 | 0 | 2 | 1 | 0 | 0 | 0 | 0 | 0 | 13 | 1.08 | 35 |
| 5279 | 2 | 1 | 0 | 0 | 1 | 1 | 0 | 1 | 0 | 0 | 0 | 0 | 0 | 4 | 0.33 | 12 |
| 4863 | 2 | 2 | 1 | 0 | 2 | 0 | 0 | 0 | 0 | 0 | 0 | 0 | 0 | 5 | 0.08 | 14 |
| 8776 | 2 | 1 | 0 | 1 | 1 | 1 | 0 | 1 | 0 | 0 | 0 | 0 | 0 | 5 | 0.08 | 11 |
| 6392 | 2 | 1 | 0 | 2 | 2 | 0 | 0 | 0 | 0 | 0 | 0 | 0 | 0 | 5 | 0.08 | 7 |
| 4773 | 2 | 4 | 0 | 0 | 3 | 1 | 0 | 0 | 0 | 0 | 0 | 0 | 0 | 8 | 0.67 | 22 |
| 9862 | 2 | 2 | 0 | 3 | 1 | 0 | 3 | 2 | 1 | 0 | 0 | 0 | 0 | 12 | 1.00 | 19 |
| 4106 | 2 | 1 | 4 | 2 | 3 | 1 | 0 | 0 | 0 | 0 | 0 | 0 | 0 | 11 | 0.92 | 25 |
| 1198 | 2 | 3 | 3 | 2 | 2 | 2 | 0 | 0 | 0 | 0 | 0 | 0 | 0 | 12 | 0.67 | 67 |
| 1376 | 2 | 1 | 3 | 1 | 2 | 0 | 0 | 0 | 0 | 0 | 0 | 0 | 0 | 7 | 0.58 | 15 |
| 7310 | 2 | 2 | 4 | 1 | 1 | 0 | 0 | 0 | 0 | 0 | 0 | 0 | 0 | 8 | 0.67 | 26 |
| 2567 | 2 | 4 | 3 | 2 | 3 | 0 | 0 | 0 | 0 | 0 | 0 | 0 | 0 | 12 | 1.00 | 47 |
| 6937 | 2 | 3 | 3 | 2 | 1 | 0 | 0 | 0 | 0 | 0 | 0 | 0 | 0 | 9 | 0.75 | 63 |
| 5463 | 3 | 2 | 2 | 0 | 0 | 1 | 1 | 0 | 2 | 1 | 0 | 1 | 1 | 11 | 1.00 | 50 |
| 1020 | 3 | 2 | 2 | 0 | 0 | 0 | 0 | 1 | 0 | 0 | 2 | 1 | 1 | 9 | 0.75 | 34 |
| 9716 | 3 | 2 | 0 | 1 | 0 | 2 | 0 | 1 | 0 | 0 | 2 | 0 | 1 | 9 | 0.75 | 20 |
| 1885 | 3 | 1 | 2 | 2 | 0 | 2 | 1 | 0 | 0 | 1 | 0 | 1 | 0 | 10 | 0.83 | 24 |
| 8992 | 3 | 2 | 1 | 2 | 0 | 3 | 0 | 2 | 1 | 0 | 2 | 1 | 1 | 15 | 1.25 | 36 |
| 1939 | 3 | 3 | 0 | 1 | 1 | 1 | 2 | 0 | 1 | 1 | 1 | 0 | 2 | 13 | 1.08 | 43 |
| 7647 | 3 | 2 | 2 | 2 | 1 | 0 | 1 | 2 | 0 | 2 | 2 | 0 | 2 | 16 | 1.33 | 65 |
| 9327 | 3 | 3 | 1 | 1 | 2 | 3 | 2 | 2 | 1 | 2 | 2 | 1 | 0 | 20 | 1.67 | 115 |
| 3993 | 3 | 4 | 3 | 0 | 2 | 0 | 3 | 2 | 3 | 3 | 2 | 0 | 1 | 23 | 1.92 | 130 |
| 5201 | 3 | 2 | 1 | 2 | 3 | 1 | 2 | 2 | 0 | 2 | 0 | 2 | 0 | 17 | 1.42 | 71 |
| 512 | 3 | 5 | 1 | 3 | 3 | 3 | 3 | 2 | 1 | 4 | 2 | 2 | 3 | 32 | 2.67 | 170 |
| 6690 | 3 | 4 | 3 | 3 | 2 | 5 | 1 | 2 | 6 | 2 | 1 | 0 | 2 | 31 | 2.75 | 163 |
| 5044 | 3 | 3 | 3 | 3 | 2 | 2 | 3 | 4 | 3 | 2 | 4 | 2 | 0 | 31 | 2.75 | 170 |
| 1138 | 3 | 6 | 3 | 2 | 3 | 3 | 4 | 2 | 2 | 4 | 3 | 3 | 2 | 37 | 3.08 | 223 |
| 4492 | 4 | 4 | 3 | 3 | 5 | 7 | 7 | 4 | 4 | 5 | 3 | 5 | 2 | 52 | 4.33 | 390 |
| 7836 | 4 | 3 | 4 | 1 | 4 | 5 | 4 | 6 | 4 | 5 | 5 | 6 | 4 | 51 | 4.25 | 186 |
| 6742 | 4 | 5 | 7 | 7 | 6 | 6 | 3 | 7 | 4 | 6 | 5 | 4 | 4 | 64 | 5.33 | 612 |
| 4996 | 4 | 3 | 5 | 5 | 7 | 5 | 7 | 3 | 7 | 8 | 7 | 5 | 6 | 68 | 5.67 | 519 |
| 5289 | 4 | 3 | 0 | 0 | 3 | 5 | 4 | 4 | 5 | 4 | 6 | 5 | 6 | 45 | 3.75 | 638 |
| 9012 | 4 | 6 | 7 | 6 | 7 | 4 | 6 | 5 | 4 | 4 | 2 | 2 | 4 | 57 | 4.75 | 333 |
| 5828 | 4 | 3 | 1 | 4 | 8 | 3 | 3 | 3 | 4 | 5 | 4 | 2 | 4 | 44 | 3.67 | 341 |
| 3985 | 4 | 2 | 6 | 4 | 4 | 7 | 6 | 4 | 6 | 7 | 5 | 6 | 6 | 63 | 5.25 | 602 |
| 3266 | 4 | 5 | 7 | 7 | 7 | 7 | 7 | 7 | 7 | 7 | 6 | 7 | 6 | 80 | 6.67 | 754 |

^a^ Level A represents participant classification: 1 = with intention but never use (n=11), 2 = dropouts (n=23), 3 = low frequency (n=14), 4 =high frequency (n=9).

^b^ Mean days is total days divided by 12 weeks.

^c^ Total behavior is all of user’s app usage behavior in 3 months, including inputting blood glucose, inputting diet, inputting weight, inputting steps, inputting drugs, reading health knowledge, signing in, and using other functions of the app.
