# Supplementary material for: Influence of Personality on mHealth Use in Patients with Diabetes: Prospective Pilot Study
Source: JMIR Mhealth Uhealth. 2020 Aug 10;8(8):e17709. doi: 10.2196/17709 (PMC7445619; doi:10.2196/17709)
Supplement: Multimedia Appendix 2 [file mhealth_v8i8e17709_app2.docx]

# Multimedia Appendix 2

Table. The detail descriptive statistics analysis for each category.

| Variables | Participants | | | Intention to use | | | |
| --- | --- | --- | --- | --- | --- | --- | --- |
|  | All | No intention to use | Intention to use | With intention but never use | Dropouts | Low frequency | High frequency |
|  |  |  |  |  |  |  |  |
| **Baseline** | | | | | | | |
| N | 98 | 41 | 57 | 11 | 23 | 14 | 9 |
| Age (years), mean (SD) | 59.23 (9.05) | 64.19 (6.70) | 55.38 (8.55) | 53.64 (7.20) | 54.96 (8.76) | 54.89 (9.28) | 60.78 (10.01) |
| Female, n(%) | 37 (38) | 18 (44) | 19 (33) | 5 (45) | 7 (30) | 6 (32) | 1 (5) |
| **Education, n(%)** |  |  |  |  |  |  |  |
| Primary/Junior | 33 (34) | 19 (46) | 14 (25) | 4 (36) | 1 (4) | 6 (43) | 3 (33) |
| Senior/Vocational | 43 (44) | 18 (44) | 25 (44) | 6 (55) | 14 (61) | 3 (21) | 2 (22) |
| Higher/University | 22 (22) | 4 (10) | 18 (31) | 1 (9) | 8 (35) | 5 (36) | 4 (44) |
| BMI (kg/m^2^), mean (SD) | 28.80 (3.19) | 25.61 (3.12) | 25.94 (3.26) | 27.22 (3.04) | 25.96 (3.08) | 25.51 (3.48) | 25.01 (3.68) |
| Disease duration (years), mean (SD) | 9.15 (8.08) | 9.44 (8.97) | 8.95 (7.45) | 9.36 (8.68) | 8.652 (7.07) | 8.71 (6.23) | 10.44 (8.59) |
| Baseline-HbA_1c_ (%), mean (SD) | 7.16 (1.00) | 7.23 (1.15) | 7.11 (0.89) | 6.95 (0.53) | 7.03 (0.97) | 7.42 (1.05) | 7.01 (0.72) |
| **Personality traits, mean (SD)** |  |  |  |  |  |  |  |
| Extraversion | 4.52 (1.70) | 4.99 (1.43) | 4.18 (1.85) | 4.91 (1.91) | 4.33 (1.90) | 3.54 (1.75) | 3.94 (1.70) |
| Agreeableness | 5.18 (1.13) | 5.21 (1.16) | 5.17 (1.12) | 4.91 (1.00) | 5.20 (1.04) | 5.21 (1.27) | 5.33 (1.37) |
| Conscientiousness | 4.63 (1.26) | 4.46 (1.15) | 4.75 (1.34) | 4.27 (1.39) | 4.78 (1.41) | 5.07 (1.22) | 4.72 (1.35) |
| Emotional stability | 4.39 (1.46) | 4.44 (1.35) | 3.36 (1.55) | 3.73 (1.57) | 4.74 (1.44) | 4.54 (1.12) | 3.89 (2.16) |
| Openness | 4.33 (1.23) | 3.93 (0.94) | 4.61 (1.34) | 4.18 (1.47) | 4.46 (1.16) | 4.82 (1.50) | 5.22 (1.30) |
| **After 3 months** | | | | | | | |
| N | 66 | 30 | 36 | 4 | 13 | 10 | 9 |
| Baseline-HbA_1c_ (%), mean (SD) | 7.14 (0.98) | 7.26 (1.13) | 7.04 (0.84) | 6.94 (0.41) | 6.93 (0.89) | 7.28 (1.02) | 7.01 (0.72) |
| Post-HbA_1c_ (%), mean (SD) | 6.84 (1.09) | 7.00 (1.26) | 6.71 (0.92) | 6.76 (1.02) | 7.01 (0.81) | 6.73 (1.09) | 6.26 (0.69) |
