# Supplementary material for: Influence of Personality on mHealth Use in Patients with Diabetes: Prospective Pilot Study
Source: JMIR Mhealth Uhealth. 2020 Aug 10;8(8):e17709. doi: 10.2196/17709 (PMC7445619; doi:10.2196/17709)
Supplement: Multimedia Appendix 3 [file mhealth_v8i8e17709_app3.docx]

# Multimedia Appendix 3

Table. Ordered logistic regression for participants (N=98).

| Variables | | β | OR | 95% CI | *P* value |
| --- | --- | --- | --- | --- | --- |
|  | |  |  |  |  |
| Age | | -0.07 | 0.93 | 0.88-0.98 | .009 |
| Female vs. male | | -0.35 | 0.71 | 0.31-1.62 | .41 |
| **Education** | |  |  |  |  |
|  | Senior vs. Primary | -0.31 | 0.73 | 0.27-1.96 | .54 |
|  | Higher vs. Primary | 0.30 | 1.35 | 0.41-4.42 | .62 |
| BMI | | -0.10 | 0.91 | 0.79-1.04 | .16 |
| Disease duration | | 0.05 | 1.05 | 0.99-1.11 | .11 |
| Baseline-HbA_1c_ | | -0.32 | 0.73 | 0.48-1.11 | .14 |
| **Personality traits** | |  |  |  |  |
|  | Extraversion | -0.35 | 0.71 | 0.55-0.91 | .007 |
|  | Agreeableness | -0.07 | 0.93 | 0.62-1.39 | .73 |
|  | Conscientiousness | 0.08 | 1.08 | 0.77-1.51 | .64 |
|  | Emotional stability | -0.02 | 0.98 | 0.73-1.31 | .88 |
|  | Openness | 0.53 | 1.69 | 1.18-2.43 | .004 |
| /Cut 1 | | -8.484 | -8.48 | -15.78 to -1.19 |  |
| /Cut 2 | | -7.862 | -7.86 | -13.98 to -0.60 |  |
| /Cut 3 | | -6.458 | -6.46 | -13.70 to 0.78 |  |
| /Cut 4 | | -5.082 | -5.08 | -12.33 to 2.17 |  |
| Chi-square (*df*) | | 37.6 (12) | | | <.001 |
| 0= No intention to use  1= With intention but never use  2= dropouts  3= low-frequency  4= high-frequency | | | | | |
