# Supplementary material for: Influence of Personality on mHealth Use in Patients with Diabetes: Prospective Pilot Study
Source: JMIR Mhealth Uhealth. 2020 Aug 10;8(8):e17709. doi: 10.2196/17709 (PMC7445619; doi:10.2196/17709)
Supplement: Multimedia Appendix 4 [file mhealth_v8i8e17709_app4.docx]

# Multimedia Appendix 4

Table. Model estimates predicting the days of app usage in adopters (n=46).

| Variables | | β | SE | *t* test | *P* value |
| --- | --- | --- | --- | --- | --- |
|  | |  |  |  |  |
| Age | | 0.05 | 0.03 | 1.45 | .16 |
| Female vs male | | -0.80 | 0.61 | -1.32 | .20 |
| **Education** | |  |  |  |  |
|  | Senior vs. Primary | -1.21 | 0.70 | -1.71 | .09 |
|  | Higher vs. Primary | -0.68 | 0.72 | -0.94 | .35 |
| BMI | | -0.06 | 0.09 | -0.64 | .53 |
| Disease duration | | 0.01 | 0.04 | 0.35 | .73 |
| Baseline-HbA_1c_ | | -0.11 | 0.30 | -0.36 | .72 |
| **Personality traits** | |  |  |  |  |
|  | Extraversion | -0.11 | 0.17 | -0.66 | .51 |
|  | Agreeableness | 0.27 | 0.26 | 1.06 | .30 |
|  | Conscientiousness | -0.32 | 0.22 | -1.48 | .15 |
|  | Emotional stability | -0.10 | 0.19 | -0.50 | .62 |
|  | Openness | 0.55 | 0.20 | 2.70 | .01 |
| Constant | | 0.45 | 5.47 | 0.08 | .93 |
| *R*^2^ | | 0.35 |  |  |  |
| *F (12, 33)* | | 1.51 | | | .17 |
