# Supplementary material for: Influence of Personality on mHealth Use in Patients with Diabetes: Prospective Pilot Study
Source: JMIR Mhealth Uhealth. 2020 Aug 10;8(8):e17709. doi: 10.2196/17709 (PMC7445619; doi:10.2196/17709)
Supplement: Multimedia Appendix 5 [file mhealth_v8i8e17709_app5.docx]

# Multimedia Appendix 5

Table. Ordered logistic regression for adopters (n=46).

| Variables | | β | OR | 95% CI | *P* value |
| --- | --- | --- | --- | --- | --- |
|  | |  |  |  |  |
| Age | | 0.07 | 1.07 | 0.97-1.17 | .14 |
| Female vs male | | -0.16 | 0.85 | 0.17-4.40 | .85 |
| **Education** | |  |  |  |  |
|  | Senior vs. Primary | -2.82 | 0.06 | 0.01-0.41 | .004 |
|  | Higher vs. Primary | -1.93 | 0.15 | 0.02-0.87 | .04 |
| BMI | | -0.11 | 0.89 | 0.70-1.14 | .36 |
| Duration | | 0.04 | 1.04 | 0.93-1.16 | .51 |
| Baseline-HbA_1c_ | | -0.05 | 0.96 | 0.44-2.08 | .91 |
| **Personality traits** | |  |  |  |  |
|  | Extraversion | -0.46 | 0.63 | 0.38-1.06 | .08 |
|  | Agreeableness | 0.37 | 1.45 | 0.73-2.86 | .29 |
|  | Conscientiousness | -0.25 | 0.78 | 0.44-1.38 | .39 |
|  | Emotional stability | -0.30 | 0.74 | 0.43-1.27 | .27 |
|  | Openness | 0.80 | 2.22 | 1.21-4.06 | .01 |
| /Cut 1 | | 0.35 | 0.35 | -13.36 to 14.07 |  |
| /Cut 2 | | 2.46 | 2.46 | -11.27 to 16.18 |  |
| Chi-square (*df*) | | 23.0 (12) | | | .03 |
| 0=dropouts  1=low-frequency  2=high-frequency | | | | | |
