# Supplementary material for: Influence of Personality on mHealth Use in Patients with Diabetes: Prospective Pilot Study
Source: JMIR Mhealth Uhealth. 2020 Aug 10;8(8):e17709. doi: 10.2196/17709 (PMC7445619; doi:10.2196/17709)
Supplement: Multimedia Appendix 6 [file mhealth_v8i8e17709_app6.docx]

# Multimedia Appendix 6

Table. Model estimates predicting changes in HbA_1c_ for all participants by the categories (n=66).

| Variables | | β | SE. | *t* test | *P* value |
| --- | --- | --- | --- | --- | --- |
|  | |  |  |  |  |
| Age | | 0.01 | 0.02 | 0.49 | .62 |
| Female vs. male | | -0.13 | 0.26 | -0.51 | .62 |
| **Education** | |  |  |  |  |
|  | Senior vs. Primary | -0.08 | 0.27 | -0.28 | .78 |
|  | Higher vs. Primary | -0.07 | 0.35 | -0.20 | .85 |
| BMI | | 0.03 | 0.04 | 0.73 | .47 |
| Duration | | 0.02 | 0.02 | 1.08 | .29 |
| Baseline-HbA_1c_ | | -0.25 | 0.13 | -1.93 | .06 |
| **Personality traits** | |  |  |  |  |
|  | Extraversion | -0.12 | 0.09 | -1.33 | .19 |
|  | Agreeableness | 0.07 | 0.13 | 0.52 | .61 |
|  | Conscientiousness | -0.08 | 0.10 | -0.88 | .38 |
|  | Emotional stability | -0.03 | 0.08 | -0.38 | .71 |
|  | Openness | 0.07 | 0.11 | 0.64 | .53 |
| 0 vs 4 | | 0.75 | 0.37 | 2.02 | .049 |
| 1 vs 4 | | 1.04 | 0.60 | 1.74 | .09 |
| 2 vs 4 | | 1.12 | 0.42 | 2.67 | .01 |
| 3 vs 4 | | 0.46 | 0.42 | 1.10 | .28 |
| Constant | | -0.05 | 2.54 | -0.02 | .99 |
| *R*^2^ | | 0.27 |  |  |  |
| *F (16, 49)* | | 1.10 | | | .38 |
| 0= No intention to use  1= With intention but never use  2= dropouts  3= low-frequency  4= high-frequency | | | | | |
